# Minimal important difference and patient acceptable symptom state for pain, Constant-Murley score and Simple Shoulder Test in patients with subacromial pain syndrome

**DOI:** 10.1186/s12874-021-01241-w

**Published:** 2021-03-06

**Authors:** Kari Kanto, Tuomas Lähdeoja, Mika Paavola, Pasi Aronen, Teppo L. N. Järvinen, Jarkko Jokihaara, Clare L. Ardern, Teemu V. Karjalainen, Simo Taimela

**Affiliations:** 1 Finnish Centre for Evidence-Based Orthopaedics (FICEBO), Department of Orthopaedics and Traumatology, Tampere University Hospital, TAYS Hatanpää, Hatanpäänkatu 24, 33900 Tampere, Finland; 2 Finnish Centre for Evidence-Based Orthopaedics (FICEBO), Department of Orthopaedics and Traumatology, University of Helsinki and Helsinki University Hospital, Topeliuksenkatu 5, HUS, 00029 Helsinki, Finland; 3 Biostatistics Unit, Faculty of Medicine at University of Helsinki and Helsinki University Hospital, Tukholmankatu 8B, 00290 Helsinki, Finland; 4 Finnish Centre for Evidence-Based Orthopaedics (FICEBO), Department of Hand and Microsurgery, Tampere University Hospital, Elämänaukio 2, 33520 Tampere, Finland; 5 Finnish Centre for Evidence-Based Orthopaedics (FICEBO), Division of Physiotherapy, Karolinska Institute, H1 Fysioterapi, 17177 Stockholm, Sweden; 6 Finnish Centre for Evidence-Based Orthopaedics (FICEBO), Central Finland Central Hospital, Jyväskylä, Keskussairaalantie 19, 40620 Jyväskylä, Finland

**Keywords:** Clinimetrics, minimal important change, MID, MCID, patient accepted symptom state, PASS, responsiveness, Outcome measures, Visual analogue scale (VAS), Simple shoulder test, Constant-Murley score, Pain, Subacromial pain

## Abstract

**Background:**

The results of clinical trials should be assessed for both statistical significance and importance of observed effects to patients. Minimal important difference (MID) is a threshold denoting a difference that is important to patients. Patient acceptable symptom state (PASS) is a threshold above which patients feel well.

**Objective:**

To determine MID and PASS for common outcome instruments in patients with subacromial pain syndrome (SAPS).

**Methods:**

We used data from the FIMPACT trial, a randomised controlled trial of treatment for SAPS that included 193 patients. The outcomes were shoulder pain at rest and on arm activity, both measured with the 0–100 mm visual analogue scale (VAS), the Constant-Murley score (CS), and the Simple Shoulder Test (SST). The transition question was a five-point global rating of change. We used three anchor-based methods to determine the MID for improvement: the receiver operating characteristic (ROC) curve, the mean difference of change and the mean change methods. For the PASS, we used the ROC and 75th percentile methods and calculated estimates using two different anchor question thresholds.

**Results:**

Different MID methods yielded different estimates. The ROC method yielded the smallest estimates for MID: 20 mm for shoulder pain on arm activity, 10 points for CS and 1.5 points for SST, with good to excellent discrimination (areas under curve (AUCs) from 0.86 to 0.94). We could not establish a reliable MID for pain at rest. The PASS estimates were consistent between methods. The ROC method PASS thresholds using a conservative anchor question threshold were 2 mm for pain at rest, 9 mm for pain on activity, 80 points for CS and 11 points for SST, with AUCs from 0.74 to 0.83.

**Conclusion:**

We recommend the smallest estimate from different methods as the MID, because it is very unlikely that changes smaller than the smallest MID estimate are important to patients: 20 mm for pain VAS on arm activity, 10 points for CS and 1.5 points for SST. We recommend PASS estimates of 9 mm for pain on arm activity, 80 points for CS, and 11 points for SST.

**Trial registration:**

ClinicalTrials.gov NCT00428870 (first registered January 29, 2007).

**Supplementary Information:**

The online version contains supplementary material available at 10.1186/s12874-021-01241-w.

## Background

The efficacy of interventions is often measured as the mean difference between intervention and control groups, or the differences in proportions of patients who achieve a desired state. It is important to judge whether a difference is important to patients, instead of relying on statistical significance testing to draw conclusions about the importance of research results. To assess whether a desired state has been achieved for an individual patient, continuous outcomes must be dichotomised to “success” and “non-success”. In most orthopaedic conditions, the outcomes most important to patients are assessed with patient-reported outcome measures (PROMs), which measure pain, function or (disease-related) quality of life. Important questions remain about their interpretation. In particular, how a certain change in PROM score is perceived by the patients, or at what level of a PROM patients consider themselves well. Concepts like MID and PASS have been developed to better understand how PROM scores reflect patients’ perceptions of their pain or disability.

The minimal important difference, MID [1], reflects the threshold at which a difference in a continuous outcome is important to patients, either between groups receiving different treatments or within-group at different time points. It is the smallest difference in the outcome of interest that informed patients or informed proxies perceive important enough to convince the patient and/or clinician to choose one treatment over another [2]. MID is commonly used to help interpret the clinical importance of the results of a trial or a meta-analysis and inform calculations of numbers needed to treat (NNTs) and sample size estimation. The MID is commonly calculated using intra-individual change in outcomes over time by anchor-based methods. In the anchor-based approach, the MID is established by relating a difference in PROM scores to a small, but important improvement or deterioration captured by an independent measure (external anchor) that is itself, interpretable. MID estimates for a given outcome can vary depending on assessment methods and patient populations [3–6].

Another measure that can help to interpret study results is the patient acceptable symptom state (PASS). The PASS is the highest symptom level at which patients consider themselves well [7]. Improving by at least MID measures “feeling better,” whereas reaching the level of at least PASS reflects “feeling good.” A patient reaching PASS would typically indicate therapeutic success at the individual level. PASS provides a tool for standardising responder rates in clinical trials. The MID and PASS concepts are complementary. For example, with values MID of 20 mm and PASS of 20 mm, if an intervention leads to a decrease of pain from VAS 80 mm to VAS 50 mm, the change is important to the patient (concept of MID) but the patient did not reach a satisfactory state (concept of PASS). Results of a trial could be expressed both as a proportion of improved patients and of patients in a satisfactory state. The definition of the PASS is anchored to the personal experience of the patient feeling well or not, and the PASS threshold for each outcome instrument of interest can be calculated using this answer as an external anchor.

Subacromial pain syndrome (SAPS) is the most common shoulder condition [8–10]. Despite the high prevalence of SAPS, only limited and almost exclusively low credibility data exist on MID and PASS thresholds of outcome instruments in patients with this condition [11]. To our knowledge, two PASS estimates for generic pain have been published for patients with SAPS [12, 13]. In other shoulder conditions, namely patients undergoing shoulder arthroplasty [13, 14] and patients with rheumatoid arthritis who are awaiting surgery [15], PASS estimates have been published for the American shoulder and elbow surgeons (ASES) score, the Simple Shoulder Test (SST), Shoulder Pain and Disability Index (SPADI), and the Visual Analog Scale (VAS) pain score.

We used the 2-year follow-up data from the FIMPACT trial [16] to estimate MID and PASS thresholds for four common shoulder outcome instruments used with patients with SAPS. We employed multiple established methods and data from a relatively large, well established, and uniform patient sample. The outcomes included three patient reported outcome measures – shoulder pain at rest, shoulder pain on arm activity (both measured using the visual analogue scale) and the Simple Shoulder Test [17]. The Constant-Murley score [18] consists of patient-reported and outcome assessor-measured components.

## Materials and methods

### Data source and study population

FIMPACT is a randomised, placebo-surgery controlled three-arm efficacy trial of subacromial decompression for treating SAPS. The trial was conducted at three orthopaedic clinics in Finland. One hundred ninety-three patients aged 35 to 65 years with SAPS were randomised to arthroscopic subacromial decompression (ASD), diagnostic arthroscopy (DA) or exercise therapy (ET), and followed for 24 months. At the eligibility screening visit, an experienced shoulder surgeon examined the patients to rule out shoulder instability, rotator cuff rupture, frozen shoulder or other causes of shoulder symptoms. All potentially eligible participants had standard x-rays and MRI to rule out rotator cuff rupture and other shoulder pathology. Baseline characteristics of participants are presented in Table S1 in the supplementary appendix and full details of the study can be found in the original articles [16, 19].

### Data time points

Pain and global rating of change (GRC) were collected at baseline, 6-, 12- and 24-month follow-ups; SST and Constant-Murley score were measured at baseline, 6- and 24-month follow-ups.

### Outcome instruments of interest

#### Pain at rest and pain on arm activity

Shoulder pain intensity during the previous 24 h was assessed on a 100 mm visual analogue scale (VAS) ranging from 0 (no pain) to 100 (extreme pain) (Fig. S1 in the supplementary appendix). Pain at rest and on arm activity were measured separately.

#### Shoulder function instruments Constant-Murley score and Simple Shoulder Test

The Constant-Murley score [18] comprises measures of capacity (range of motion and strength) and subjective parameters (pain assessment, work load, and leisure time activities), which yield a score ranging from 0 (worst) to 100 (best). Although the Constant-Murley score is one of the most frequently cited instruments, it does not have convincing evidence for its psychometric properties [20].

The Simple Shoulder Test (SST) [17], consists of 12 questions of shoulder status and function, with yes (1) or no (0) response options. Answers are summed for a score ranging from 0 to 12, with maximum score indicating normal shoulder function. The Simple Shoulder Test has good evidence in support of internal consistency, reliability, structural validity, hypothesis testing, and responsiveness [20].

#### Global rating of change

Participants were asked their subjective satisfaction to treatment outcome relative to baseline at the 6-, 12- and 24-month follow-up visits on a five-point global rating of change (GRC) scale (Table 1).

**Table 1:** Global Rating of Change response options^a^

1. Very satisfied—my shoulder has healed completely.	
2. Satisfied—I have only minor, activity related symptoms. My shoulder is much better than before treatment.	
3. Somewhat satisfied—I have only minor symptoms. My shoulder is better than before treatment.	
4. Dissatisfied—my shoulder is the same as before treatment.	
5. Very dissatisfied—my shoulder is worse than before treatment.	

^a^Participants were instructed to choose the answer that best represented their current situation. The answer options 1–5 have been translated from Finnish to English

### Data analysis for MID

We used the GRC as the anchor question for calculating the MID. An adequate transition anchor should correlate to the change in outcome, and ideally correlate equally, but in opposite directions to the scores of outcomes at baseline and at follow-up time points (post scores) [21]. The correlation to change should be larger than the correlation to post scores when the GRC captures true change [22]. To explore this, we calculated the correlation coefficients (Spearman’s rho) for the GRC answers at different time points and baseline scores, the GRC and each of the respective post scores and the post scores of the combined dataset, and the GRC and the change scores of the outcomes, also at follow-up time points and the combined dataset. 95% CIs were calculated by bootstrapping 1000 samples for the correlations between the anchor and relevant scores.

We used three approaches to determine the MID for improvement: 1) the ROC method, 2) the mean difference of change (MDoC) method and 3) the mean change (MC) method.

For the ROC method [23], we dichotomised the GRC to improved (responses 1–3; Table 1) and no change (response 4; Table 1). Participants with response worse (response 5; Table 1) were excluded from the ROC analyses to obtain MID estimates for improvement [24]. Because very few patients deteriorated, we could not estimate MIDs for worsening. We used the closest point to top left corner method to choose the cut-off value for the outcome, maximising specificity and sensitivity [25]. For the target measures, we calculated change from baseline to each follow-up point.

To evaluate how well each measure could discriminate between those who were improved and those who were not improved, we calculated the area under the ROC curve (AUC). We determined the confidence intervals for AUC using DeLong’s method [26]. The area ranges from 0.5 (no accuracy in distinguishing improved from not improved) to 1.0 (perfect accuracy) [27, 28]. In musculoskeletal conditions, AUC values between 0.7 and 0.8 are acceptable, and value greater than 0.8 is considered to have good to excellent discrimination [29].

In the MDoC method, we calculated the mean difference of the change scores of each outcome from baseline to the follow-up time point (with 95% CIs) between the participants who answered” Somewhat satisfied” and” Dissatisfied” (responses 3 and 4; Table 1). In the MC method, we determined the mean of the change scores from baseline to the follow-up time points (with 95% CIs) of those who reported” Somewhat satisfied” (response 3; Table 1). With the MDoC and MC methods, the 95% CIs were calculated by bootstrapping 1000 samples for the MID values.

We combined the data across all time points (6, 12, 24 months) and used the whole dataset irrespective of treatment for analyses to provide an estimate derived from a larger number of GRC-outcome pairs. We explored the ROC curves, and MID and PASS estimates at different time points and found them to be very similar, supporting our decision to pool data for our primary analysis. To explore whether the different treatments affected the MIDs, we performed sensitivity analyses and calculated MIDs for patients who underwent surgery (ASD and DA groups combined) and for patients who received exercise therapy. In the FIMPACT trial, the blinding between ASD and DA held well, and the patients in both ASD and DA groups subjectively underwent “surgical treatment”.

### Data analysis for PASS

For PASS, we used the ROC and the 75th percentile [30] methods for the combined dataset. The ROC method was used similarly as in MID. We used the closest point to top left corner method [25] to determine the cut-off point and the AUCs were used to evaluate how well each measure could discriminate between participants who reported “Very satisfied, my shoulder has healed completely” and the rest of the cohort (responses 2–5, Table 1). In 75th percentile method, PASS was defined as the 25th percentile score for Constant-Murley score and Simple Shoulder Test, and 75th percentile score for pain VASs from the distribution of the patients who answered: “Very satisfied, my shoulder has healed completely”. Because the choice whether to use GRC 1 only or both 1 and 2 is debatable, we also calculated the PASS thresholds between participants who reported “Very satisfied, my shoulder has healed completely” and “Satisfied—I have only minor, activity related symptoms. My shoulder is much better than before treatment.” (responses 1–2, Table 1) and the rest of the cohort (responses 3–5, Table 1).

### Compliance with research ethics standards

This study was conducted in compliance with good clinical practice, and the Declaration of Helsinki.

## Results

We analysed data from 193 patients. Due to missing data items, the number of available GRC-outcome pairs varied at each time point (Table 2).

**Table 2:** Numbers of data pairs

	6 months	12 months	24 months	Combined
Pain at rest	179	176	184	539
Pain on arm activity	179	175	184	538
Constant-Murley score	179	–	179	358
Simple Shoulder Test	179	–	180	359

- denotes that outcomes were not measured at the time point

To test the adequacy of our transition anchor, we calculated the correlations to the baseline values and outcomes at each time point. The correlations between the GRC and baseline values were close to zero (Table S2 in the supplementary appendix). The correlations between the GRC and post scores in the combined data were for pain at rest − 0.57 (− 0.63 to − 0.50), for pain on activity − 0.63 (− 0.69 to − 0.56), for Constant-Murley score 0.63 (0.55 to 0.69) and for Simple Shoulder Test 0.62 (− 0.55 to 0.69). The correlations between the GRC and change scores in the combined data were for pain at rest − 0.25 (− 0.32 to − 0.16), for pain on activity − 0.53 (− 0.59 to − 0.47), for Constant-Murley score 0.57 (0.49 to 0.64) and for Simple Shoulder test 0.49 (0.40 to 0.57). The correlations to post scores and change scores at individual time points are presented in Table S2 in the supplementary appendix. The correlation between GRC and change of pain at rest was very low, whereas the correlations between the GRC and change of other outcomes were adequate. The correlations to post scores were larger than the correlations to change.

### MID estimates

#### MIDs based on receiver operating characteristic method

MID estimates from the ROC analysis with their characteristics are presented in Table 3. In the ROC analysis, MID for Constant-Murley score had excellent discrimination (AUC), while MIDs for pain on arm activity and SST showed good discrimination. Discrimination improved with the follow-up time (Table S3 in the supplementary appendix). MID for pain at rest did not discriminate well. The ROC curves are presented in Fig. S2 in the supplementary appendix.

**Table 3:** MID estimates from the ROC analysis

Outcome measure	MID	Sensitivity	Specificity	AUC (95% CI)
Pain at rest (VAS 0–100)	13	0.63	0.72	0.72 (0.64 to 0.81)
Pain on arm activity (VAS 0–100)	20	0.90	0.78	0.86 (0.82 to 0.91)
Constant-Murley score (0–100)	10	0.90	0.89	0.94 (0.92 to 0.97)
Simple Shoulder Test (0–12)	1.5	0.83	0.78	0.87 (0.82 to 0.92)

#### MIDs based on mean difference of change and mean change methods

MIDs with 95% CIs based on the MDoC and MC methods are presented in Table 4. MID values with 95% CIs from data at each timepoint can be found in the Table S4 in the supplementary appendix.

**Table 4:** MID estimates from the mean difference of change (MDoC) and mean change (MC) analyses

Outcome measure	Method	MID (95% CI)
Pain at rest (VAS 0–100)	MDoC	16 (7 to 27)
	MC	23 (18 to 27)
Pain on arm activity (VAS 0–100)	MDoC	24 (14 to 34)
	MC	26 (20 to 32)
Constant-Murley score (0–100)	MDoC	24 (18 to 31)
	MC	23 (19 to 27)
Simple Shoulder Test (0–12)	MDoC	2.3 (1.4 to 3.3)
	MC	2.7 (2.1 to 3.3)

MID values calculated by the MDoC and MC methods were larger than the MID values from the ROC analysis, especially for the Constant-Murley score by a factor of two: In the primary analysis 9.5 points with ROC method; 23 points with MDoC method; 24 points with MC method. Similar results were obtained in the analyses of separate time points. The estimates of MID values calculated from data of patients who underwent surgery were similar to MIDs derived from patients who received exercise therapy, but the confidence intervals were wide and the ROC curves were not ideal, making these subgroup results unreliable.

### PASS estimates

Estimates for PASS derived by the ROC and the 75th percentile methods are presented in Table 5. The AUCs had acceptable to good discrimination. The ROC curves are presented in Fig. S3 in the supplementary appendix. The PASS estimates provided by the 75th percentile method were almost identical to the values from the ROC analysis.

**Table 5:** PASS estimates

Outcome measure	75th percentile method	ROC method			
	PASS	PASS	Sens	Spec	AUC (95% CI)
**Pain at rest (VAS 0–100)**					
GRC answer 1	2	2	0.71	0.69	0.74 (0.70 to 0.79)
GRC answers 1 + 2	6	8	0.79	0.75	0.83 (0.79 to 0.86)
**Pain on arm activity (VAS 0–100)**					
GRC answer 1	10	9	0.80	0.75	0.83 (0.79 to 0.88)
GRC answers 1 + 2	29	26	0.73	0.77	0.82 (0.78 to 0.86)
**Constant-Murley score (0–100)**					
GRC answer 1	80	81	0.73	0.74	0.78 (0.73 to 0.83)
GRC answers 1 + 2	71	69	0.81	0.75	0.85 (0.80 to 0.89)
**Simple Shoulder Test (0–12)**					
GRC answer 1	11	11	0.68	0.81	0.80 (0.75 to 0.85)
GRC answers 1 + 2	9	9	0.81	0.70	0.83 (0.78 to 0.87)

GRC answer 1: Very satisfied—my shoulder has healed completely

GRC answer 2: Satisfied—I have only minor, activity related symptoms. My shoulder is much better than before treatment

## Discussion

Our MID estimates for pain on arm activity, the Constant-Murley score, and the Simple Shoulder Test appear trustworthy: In the ROC analysis they showed good (pain on arm activity and Simple Shoulder Test) or excellent (Constant-Murley score) discrimination between patients who considered themselves improved or not improved. The correlations to change scores were adequate for these three outcomes, but the correlations to post scores were slightly larger than the correlations to change scores. MID values derived for pain at rest do not appear useful in this patient population. When interpreting trial results, the smallest credible estimate from different methods for MID sets the low limit for the MID, as changes smaller than the smallest MID estimate are very unlikely to be important to patients. The likely best MID estimates were 20 mm for pain VAS on arm activity, 10 points for Constant-Murley score and 1.5 points for Simple Shoulder Test.

The PASS estimates using GRC 1 answer only for pain on arm activity (9 mm), Constant-Murley score (81 points), and Simple Shoulder Test (11 points) were consistent between methods and showed good discrimination. Using GRC answers 1 + 2, the PASS estimates were also consistent between methods and the AUCs were better or similar to the analysis using answer option 1 only. The PASS estimates with GRC 1 + 2 were 8 for pain at rest, 26 for pain on arm activity, 69 for the Constant-Murley score and 9 for the Simple Shoulder Test. We recommend using more conservative of the estimates for PASS values, which we think certainly represents a state of being well.

In line with previous findings [3, 4], our analysis based on the FIMPACT trial data found high variability of the MID estimates both between methods and outcome instruments. These findings showcase the challenges of the MID concept and highlight the need for deep understanding of the instruments, statistical methods, and differences in patient populations when applying the MID results in clinical practice.

### Strengths and weaknesses of the study

We used multiple established methods to estimate the MID and PASS values in a relatively large patient sample with high adherence to follow-up (92% at 24 months). Our study population was exceptionally well established and uniform: a robust clinical examination by highly experienced orthopaedic surgeons ensured that participants had clinical findings consistent with SAPS, and magnetic resonance imaging with intra-articular contrast agent (MRA) was used to exclude other shoulder pathology. The strict inclusion criteria may limit the generalisability of our results to other shoulder conditions.

The anchor question we used for determining the PASS was not verbatim the recommended PASS question [31]. The FIMPACT trial was initiated prior to the publication of the PASS concept and the recommended anchor question, forcing us to use the best available PASS anchor. The choice between GRC 1 and 1 + 2 to represent a satisfied patient is not clear, so we calculated the PASS values for both choices. We recommend the PASS estimates calculated with GRC answer 1 only, thinking that erring on the side of caution would be the wise choice here. While acknowledging that our choice can provide a conservative threshold for PASS, we are confident that patients are satisfied with a “completely healed shoulder” and that this response option truly represents a state of being well.

Given the relatively long interval between the baseline and first follow-up, risk of recall bias is obvious. This notion is supported by very low correlations between the transition item and baseline and lower correlations to change scores than to the post scores [21, 22]. This is an inherent weakness of the GRC in a setting where the condition needs longer to evolve than a reliable recall time frame [22]. The AUCs generated by the ROC method had good to excellent discrimination. Also, each of the GRC response options contained a description of satisfaction to the change (and some also a statement of state) and this could affect patient responses compared to a pure satisfaction or change questions. However, the patients were very symptomatic at the baseline, and we think that the answer options capture change in their wording, and the best category also represents a satisfactory state.

### Comparison to previous studies

The method of determining MID affected the values in our study [3, 4]. The lowest cut-offs were obtained with the ROC method (20 mm for pain on arm activity, 10 points for Constant-Murley score and 1.5 points for Simple Shoulder Test) and the highest with the MC method (26, 23, and 2.7, respectively).

A recent systematic review of anchor-based MIDs for improvement in patient-reported outcomes provided MID estimates for mixed shoulder conditions [11]. There was large variation in reported values between studies. The median estimate for MID concerning pain at rest measured with VAS was 30 mm and for pain VAS on arm activity was 21 mm [11]. Our MID estimate for pain VAS on arm activity is in line with the systematic review [11], but there is a marked difference in MID estimates for pain at rest. In our study, change scores of pain VAS at rest did not show adequate correlation with the anchor question and the AUCs in the ROC analysis were low, which in our opinion aligns well with the clinical reality that pain at rest is rarely the predominant symptom driving patients with SAPS to seek medical attention. The MID estimate for Constant-Murley score was 8.3 points in the systematic review, which is very similar to our estimate (10 points). The median estimate for SST was 1.8 points [11], again consistent with our result of 1.5 points. Another recent systematic review [32] identified two studies that had assessed MID estimates for Constant-Murley score in patients with rotator cuff tears. The MID estimates were 8 to 10 points.

We identified two studies that attempted to determine PASS estimates for VAS or numeric pain rating scale (NPRS) of patients treated for subacromial pain. Tubach et al. [12] reported PASS estimates for VAS ranging from 16 to 24 mm in patients treated nonoperatively for “acute rotator cuff syndrome.” Tashjian et al. [13] reported a PASS estimate of 30 mm for shoulder pain VAS for patients with rotator cuff disease treated without surgery. Neither study reported separate values for pain at rest or pain on arm activity. Nevertheless, our recommended estimates are markedly lower, probably due to use of only the “very satisfied, completely healed” category as the anchor for PASS.

We were not able to identify studies reporting PASS estimates for Constant-Murley score or Simple Shoulder Test in patients with subacromial pain syndrome.

### Meaning of the study

The smallest trustworthy estimate from different methods for MID can be used because anything less than the smallest MID estimate should be interpreted as unimportant to the patient. There may be settings where using the highest estimate is useful, for example, in a superiority trial where there is a large difference between treatments the higher limit for MID might be a good choice as a threshold for “unequivocal effectiveness.”. In our study, the ROC method provided the smallest estimates and discriminated well between those who considered themselves improved from those not improved. Pain at rest showed poor correlation with the anchor question and change score and low ability to discriminate, reflecting its low usefulness in this patient population.

Our PASS estimates for pain at rest and pain on arm activity, Constant-Murley score, and Simple Shoulder Test were consistent across methods and showed good to excellent discrimination between those who considered themselves well from those who did not. We chose to recommend using the estimates derived from analysis using GRC answer 1 only, but it is likely to be conservative, as the acceptable symptoms state may include some minor symptoms also. When the PASS estimates are used to interpret study results, depending on the study setting and characteristics of the patient population, the estimates derived using GRC 1 + 2 can be applicable as well.

### Unanswered questions and future research

The MID estimates vary widely, depending on assessment methods and patient populations [33]. Change appears to be baseline dependent: people with more severe symptoms need to experience a greater change to consider their condition improved [30, 34] and the results are sensitive to the time point and anchor questions used. Future research topics include determining and then standardising the best method(s) – including the anchor question – for estimating the MID. Qualitative approaches might also have a place in future research [35].

## Conclusion

Different methods provided different estimates for MIDs. We recommend MID estimates for patients with subacromial pain as follows: 20 mm for pain VAS on arm activity, 10 points for Constant-Murley score and 1.5 points for Simple Shoulder Test. We could not establish a reliable MID for changes in pain at rest in this patient population. We recommend PASS estimates of 9 mm for pain on arm activity, 80 points for Constant-Murley score, and 11 points for Simple Shoulder Test.

## Supplementary Information

**Additional file 1: Table S1**. Baseline characteristics of the participants according to study group. **Table S2** Correlations between GRC and baseline, post scores and change scores*. **Table S3** MID estimates from the ROC analysis at 6, 12, 24 months. **Table S4** MID values calculated by the mean difference of change (MDoC) and the mean change (MC) methods and their respective 95% confidence intervals (CI). **Fig. S1** VAS figure from the original questionnaire (translated from Finnish to English). **Fig. S2** MID ROC curves. **Fig. S3** PASS ROC curves.

## Data Availability

FIMPACT data are not publicly available owing to data privacy issues, but access to the anonymised dataset can be obtained from the corresponding author on reasonable request.

## Abbreviations

AUC: Area under curve
CS: Constant-Murley score
GRC: Global rating of change
MC: Mean change
MDoC: Mean difference of change
MID: Minimal important difference
NNT: Numbers needed to treat
NPRS: Numeric pain rating scale
PASS: Patient acceptable symptom state
ROC: Receiver operating characteristic
SAPS: Subacromial pain syndrome
SPADI: Shoulder Pain and Disability Index
SST: Simple Shoulder Test
VAS: Visual analogue scale
