# Supplementary material for: Minimal important difference and patient acceptable symptom state for pain, Constant-Murley score and Simple Shoulder Test in patients with subacromial pain syndrome
Source: BMC Med Res Methodol. 2021 Mar 6;21:45. doi: 10.1186/s12874-021-01241-w (PMC7937213; doi:10.1186/s12874-021-01241-w)
Supplement: Supplementary file 1 — Additional file 1: Table S1. Baseline characteristics of the participants according to study group. Table S2 Correlations between GRC and baseline, post scores and change scores*. Table S3 MID estimates from the ROC analysis at 6, 12, 24 months. Table S4 MID values calculated by the mean difference of change (MDoC) and the mean change (MC) methods and their respective 95% confidence intervals (CI). Fig. S1 VAS figure from the original questionnaire (translated from Finnish to English). Fig. S2 MID ROC curves. Fig. S3 PASS ROC curves. [file 12874_2021_1241_MOESM1_ESM.docx]

**Minimal Important Difference and Patient Acceptable Symptom State for Pain, Constant-Murley Score and Simple Shoulder Test in Patients with Subacromial Pain Syndrome**

Kari Kanto^1^, Tuomas Lähdeoja^2^, Mika Paavola^2^, Pasi Aronen^3^, Teppo L N Järvinen^2^, Jarkko Jokihaara^4^, Clare L Ardern^5^, Teemu V Karjalainen^6^ and Simo Taimela^2^

**Supplementary Appendix**

This appendix has been provided by the authors to give readers additional information about their work.

____________________________

^1^ Finnish Centre for Evidence-Based Orthopaedics (FICEBO), Department of Orthopaedics and Traumatology, Tampere University Hospital, TAYS Hatanpää, Hatanpäänkatu 24, 33900 Tampere, Finland

^2^ Finnish Centre for Evidence-Based Orthopaedics (FICEBO), Department of Orthopaedics and Traumatology, University of Helsinki and Helsinki University Hospital, Topeliuksenkatu 5, HUS 00029 Helsinki, Finland

^3^ Biostatistics Unit, Faculty of Medicine at University of Helsinki and Helsinki University Hospital, Tukholmankatu 8B, 00290 Helsinki, Finland

^4^ Finnish Centre for Evidence-Based Orthopaedics (FICEBO), Department of Hand and Microsurgery, Tampere University Hospital, Elämänaukio 2, 33520 Tampere, Finland

^5^ Finnish Centre for Evidence-Based Orthopaedics (FICEBO), Division of Physiotherapy, Karolinska Institute, H1 Fysioterapi, 17177 Stockholm, Sweden

^6^ Finnish Centre for Evidence-Based Orthopaedics (FICEBO), Central Finland Central Hospital, Jyväskylä, Keskussairaalantie 19, 40620 Jyväskylä, Finland

**Table of Contents**

Table S1 Baseline characteristics of the participants according to study group …………………………..3

Table S2 Table S2 Correlations between GRC and baseline, post scores and change scores ....…….….. .4

Table S3 MID estimates from the ROC analysis at 6, 12, 24 months .…………….….….……….………5

Table S4 MID values calculated by the mean difference of change (MDoC) and the mean change (MC) methods and their respective confidence intervals ……………………………………………..6

Fig. S1 VAS figure from the original questionnaire (translated from Finnish to English) ………….…7

Fig. S2 MID ROC curves ………………………………………………….…………...……...….....…8

Fig. S3 PASS ROC curves ………………………………….…………...……………………………..9

| **Table S1 Baseline characteristics of the participants according to study group** | | | |
| --- | --- | --- | --- |
|  | **ASD (n=59)** | **DA (n=63)** | **ET (n=71)** |
| Age, mean (SD) | 50.5 (7.3) | 50.8 (7.6) | 50.4 (6.6) |
| Sex Male, n (%) | 17 (29%) | 17 (27%) | 24 (34%) |
| Female, n (%) | 42 (71%) | 46 (73%) | 47 (66%) |
| Dominant hand affected, n (%) | 35 (59%) | 36 (57%) | 46 (65%) |
| Duration of symptoms (Months), mean (SD) | 18 (14) | 18 (19) | 22 (23) |
| Ability to work normally regardless of the shoulder symptoms? (yes), n (%) | 27 (46%) | 31 (49%) | 35 (49%) |
| VAS (at rest)^*^, mean (SD) | 41.3 (25.8) | 41.6 (25.5) | 41.7 (27.5) |
| VAS (at arm activity)^*^, mean (SD) | 71.2 (23.6) | 72.3 (21.7) | 72.4 (20.8) |
| Constant-Murley score†, mean (SD) | 32.2 (15.8) | 31.7 (14.0) | 35.2 (16.2) |
| SST score‡, mean (SD) | 4.9 (2.9) | 4.9 (2.9) | 4.8 (2.7) |
| ^*^Shoulder pain at rest and at activity was assessed on a 100 mm Visual analog scale (VAS) of 0 to 100, with 0 denoting no pain and 100 denoting extreme pain.  †Constant-Murley score (CS) is a scoring system for evaluation of various disorders of the shoulder consisting of both objective (range of motion and strength) and subjective measurements (pain assessment, work load, and leisure time activities), which are summarised in a score between 0 and 100. A higher score indicates better shoulder function.  ‡The simple shoulder test (SST) is a based on 12 questions with yes (1) or no (0) response options. The maximum SST score is 12, indicating normal shoulder function, while the minimum score of 0 points refers severely diminished shoulder function. | | | |

| **Table S2 Correlations between GRC and baseline, post scores and change scores*.** | | | | | | | |
| --- | --- | --- | --- | --- | --- | --- | --- |
|  | **Correlation to** |  | **Correlation to post score** | | **Correlation to change score** | | |
|  | **baseline** | **6 months** | **12 months** | **24 months** | **6 months** | **12 months** | **24 months** |
| **Pain at rest (VAS 0-100)** | | | | | | | |
| GRC 6 months | -0.12 (-0.26 to 0.02) | -0.58 (-0.68 to -0.46) |  |  | -0.26 (-0.39 to -0.12) |  |  |
| GRC 12 months | -0.11 (-0.24 to 0.03) |  | -0.52 (-0.63 to -0.40) |  |  | -0.24 (-0.37 to -0.09) |  |
| GRC 24 months | -0.13 (-0.26 to 0.01) |  |  | -0.54 (-0.65 to -0.42) |  |  | -0.19 (-0.32 to -0.06) |
| **Pain on activity (VAS 0-100)** | | | | | | | |
| GRC 6 months | -0.08 (-0.22 to 0.08) | -0.61 (-0.72 to -0.49) |  |  | -0.53 (-0.65 to -0.41) |  |  |
| GRC 12 months | 0.02 (-0.12 to 0.18) |  | -0.62 (-7.12 to -0.51) |  |  | -0.54 (-0.65 to -0.42) |  |
| GRC 24 months | -0.01 (-0.15 to 0.13) |  |  | -0.58 (-0.68 to -0.45) |  |  | -0.43 (-0.54 to -0.30) |
| **Constant-Murley score** | | | | | | | |
| GRC 6 months | -0.03 (-0.18 to 0.13) | 0.65 (0.55 to 0.73) |  |  | 0.64 (0.53 to 0.72) |  |  |
| GRC 12 months | N/A |  | N/A |  |  | N/A |  |
| GRC 24 months | 0.03 (-0.12 to 0.18) |  |  | 0.49 (0.36 to 0.61) |  |  | 0.40 (0.26 to 0.54) |
| **Simple Shoulder Test** | | | | | | | |
| GRC 6 months | 0.03 (-0.13 to 0.19) | 0.60 (0.49 to 0.69) |  |  | 0.56 (0.45 to 0.66) |  |  |
| GRC 12 months | N/A |  | N/A |  |  | N/A |  |
| GRC 24 months | 0.06 (-0.07 to 0.22) |  |  | 0.55 (0.42 to 0.66) |  |  | 0.32 (0.17 to 0.45) |

*Data shown as Spearman's rho (95% CI)

| **Table S3 MID estimates from the ROC analysis at 6, 12, 24 months** | | | | |
| --- | --- | --- | --- | --- |
| **Outcome measure** | **MID** | **Sensitivity** | **Specificity** | **AUC (95% CI)** |
| **6 months** | | | | |
| Pain at rest (VAS 0-100) | 12.5 | 0.65 | 0.69 | 0.68 (0.56 to 0.80) |
| Pain on activity (VAS 0-100) | 19.5 | 0.90 | 0.72 | 0.83 (0.75 to 0.91) |
| Constant-Murley score | 9.5 | 0.87 | 0.85 | 0.93 (0.88 to 0.97) |
| Simple Shoulder Test | 1.5 | 0.78 | 0.75 | 0.83 (0.74 to 0.92) |
| **12 months*** | | | | |
| Pain at rest (VAS 0-100) | 1.5 | 0.57 | 0.87 | 0.70 (0.53 to 0.87) |
| Pain on activity (VAS 0-100) | 19.0 | 0.93 | 0.79 | 0.86 (0.78 to 0.94) |
| **24 months** | | | | |
| Pain at rest (VAS 0-100) | 2.5 | 0.71 | 0.86 | 0.82 (0.62 to 0.97) |
| Pain on activity (VAS 0-100) | 11.5 | 0.86 | 0.86 | 0.91 (0.84 to 0.97) |
| Constant-Murley score | 9.5 | 0.92 | 1.00 | 0.97 (0.94 to 1.00) |
| Simple Shoulder Test | 1.5 | 0.88 | 0.86 | 0.91 (0.86 to 0.97) |

*Constant-Murley score and Simple Shoulder Test were not measured at 12 months.

| **Table S4 MID values calculated by the mean difference of change (MDoC) and the mean change (MC) methods and their respective 95% confidence intervals (CI)** | | |  |
| --- | --- | --- | --- |
| **Outcome measure** | **Method** | **MID (95% CI)** | |
| **6 months** | | | |
| Pain at rest (VAS 0-100) | MDoC | 10 (4 to 24) | |
|  | MC | 20 (12 to 27) | |
| Pain on activity (VAS 0-100) | MDoC | 22 (9 to 35) | |
|  | MC | 24 (17 to 33) | |
| Constant-Murley score | MDoC | 19 (13 to 25) | |
|  | MC | 20 (15 to 24) | |
| Simple Shoulder Test | MDoC | 1.9 (0.6 to 3.0) | |
|  | MC | 2.2 (1.6 to 2.9) | |
| **12 months*** | | | |
| Pain at rest (VAS 0-100) | MDoC | 18 (-4 to 35) | |
|  | MC | 24 (15 to 33) | |
| Pain on activity (VAS 0-100) | MDoC | 24 (9 to 37) | |
|  | MC | 29 (22 to 37) | |
| **24 months** | | | |
| Pain at rest (VAS 0-100) | MDoC | 30 (8 to 54) | |
|  | MC | 27 (19 to 35) | |
| Pain on activity (VAS 0-100) | MDoC | 28 (12 to 44) | |
|  | MC | 28 (19 to 37) | |
| Constant-Murley score | MDoC | 35 (21 to 51) | |
|  | MC | 27 (19 to 34) | |
| Simple Shoulder Test | MDoC | 3.0 (1.4 to 4.4) | |
|  | MC | 3.4 (2.4 to 4.4) | |

* Constant-Murley score and Simple Shoulder Test were not measured at 12 months.

**Fig. S1 VAS figure from the original questionnaire (translated from Finnish to English)**

**VAS / pain**

1. Please place a vertical mark on the line at the point that represents current level of pain at rest.

No _______________________________________________ Worst

pain possible

pain

1. Please place a vertical mark on the line at the point that represents current level of pain on arm activity.

No _______________________________________________ Worst

pain possible

pain

| **Fig. S2 MID ROC curves** |
| --- |
|  |

| **Fig. S3 PASS ROC curves** |
| --- |
|  |

**Fig. S3 PASS ROC curves (cont.)**
